# Maxillary Expansion: A Comparison of Damon Self-Ligating Bracket Therapy with MARPE and PAOO

**DOI:** 10.1155/2022/1974467

**Published:** 2022-05-09

**Authors:** Eman Alsayegh, Nasib Balut, Donald J. Ferguson, Laith Makki, Thomas Wilcko, Ismaeel Hansa, Nikhilesh R. Vaid

**Affiliations:** ^1^Department of Orthodontics, European University College, Dubai, UAE; ^2^Universidad Autónoma de Baja California, Mexicali, Mexico; ^3^Universidad Del Valle, Cali, Colombia; ^4^Department of Periodontology, Case Western Reserve University, Cleveland, USA

## Abstract

**Purpose:**

The aim of this study was to investigate arch parameters and dentoalveolar changes from pretreatment to posttreatment by comparing the Miniscrew Assisted Rapid Palatal Expansion (MARPE), Periodontally Accelerated Osteogenic Orthodontics (PAOO), and Damon self-ligating bracket therapies.

**Materials and Methods:**

Seventy-nine patients underwent maxillary expansion followed by or in conjunction with Damon (*n* = 23), PAOO (*n* = 28), and MARPE (*n* = 28) therapies. Nine maxillary dental arch parameters were compared at pretreatment, posttreatment as well as, increments of treatment change. Measurements were made on STL study casts using 3Shape Ortho Analyzer 3D scanner software.

**Results:**

All groups showed significant posterior width increase in the molar area. The mean increase in inter-molar distance was more than 8X greater in MARPE group compared to Damon and more than 4X greater compared to PAOO. MARPE showed significantly greater increments of change in inter-molar width and palatal vault area

**Conclusions:**

All groups showed a significant width increase in the canine and molar area. MARPE showed the greatest increase in inter-molar width, followed by PAOO and Damon. MARPE was the only group to show a significant increase in palatal vault area.

## 1. Introduction

Transverse maxillary deficiency is a frequently occurring problem in patients presenting for orthodontic treatment. Approximately 9% of the US population have a transverse maxillary deficiency associated with a posterior crossbite [1]. Transverse maxillary deficiency is a skeletal deficiency and may also cause and influence the sagittal and occlusal dimensions, such as dental protrusion and crowding. Treatment of the transverse dimension therefore plays a vital part in resolving arch perimeter problems, especially when extractions are contraindicated [2].

Conventional rapid palatal expansion (RPE) has been used as a proven method for treating transverse maxillary deficiency in pre-pubertal children. Its usefulness in post-pubertal patients however is limited, as the circum-maxillary sutures fuse, resulting in little or no skeletal effects [3]. Due to the lack of skeletal expansion and the potential for damage to the periodontium, surgically assisted rapid palatal expansion (SARPE) has traditionally been the gold standard when treating transverse maxillary deficiency in the adult patient [4]. SARPE is an invasive procedure however, and the costs, risks, and morbidity associated to the surgery may discourage many patients, and orthodontists, from seeking correction through this procedure [5].

Much attention has been given recently to less invasive expansion procedures. The ability to resolve severe malocclusion without the need for surgical intervention has tremendous potential for benefit to the patient and orthodontist [6]. Lee et al. [7] introduced the Miniscrew Assisted Rapid Palatal Expansion (MARPE), in which miniscrews are used in conjunction with an expansion appliance, and reported successful opening of the midpalatal suture. Despite the high success rate of MARPE, in older patients, where the sutures may be closely interdigitated, it may still be difficult to split the midpalatal and circum-maxillary sutures despite utilizing cortical anchorage [8].

Wilcko et al. [9] introduced the Periodontally Accelerated Osteogenic Orthodontics (PAOO) which involves alveolar decortication with bone graft augmentation, combined with orthodontic treatment. PAOO has been shown to not only expand the scope of orthodontic tooth movement by 200 to 300% in most dimensions, but also hastens tooth movement due to the Regional Acceleratory Phenomenon (RAP) [10]. Ferguson et al. [10] demonstrated that up to 7 mm of inter-canine width was attained by expansion via the arch wires alone, following labial and lingual corticotomy and bone graft augmentation extending anteriorly between the two maxillary first molars.

The conventional notion was that once skeletal maturity had been reached, orthodontic treatment alone could not offer significant nor stable expansion of the maxilla for deficiencies greater than 5 mm [11]. Birnie [12] however claimed that the Damon System, which is a passive self-ligation system, has the ability to achieve significant posterior expansion without any need for auxiliary appliances such as RPE appliances. The Damon philosophy indicates that light forces do not overpower the musculature and periodontium, but rather the arch form aligns by posterior expansion due to the lesser resistance of the musculature.

To date, there have been no investigations of the treatment effects of expansion with Damon, MARPE, or PAOO. The aim of this study was to investigate arch parameters and dentoalveolar changes from pretreatment to posttreatment by comparing MARPE, PAOO, and Damon self-ligating bracket therapies. The null hypothesis tested was no significant difference in dentoalveolar changes when using Damon self-ligating non-extraction treatment compared to PAOO and MARPE therapies.

## 2. Materials and Methods

### 2.1. Sample

This retrospective cohort study evaluated the pretreatment and posttreatment STL study casts of adult patients treated with maxillary expansion using three different orthodontic treatment modalities. Inclusion criteria were as follows: (1) moderate to severe transverse skeletal discrepancy (5 mm or more); (2) patients greater than 16 years of age; (3) presence of a posterior unilateral or bilateral cross-bite; (4) availability of pretreatment and final outcome study casts; (5) presence of all teeth anterior to, and including, the first molars; and (6) non-extraction orthodontic treatment. Exclusion criteria were as follows: (1) prior orthodontic treatment, (2) craniofacial congenital anomalies, and (3) taking any medication that might affect bone density.

### 2.2. Study Groups

A total of 79 patients fit the criteria: Damon (*n* = 23), MARPE (*n* = 28), and PAOO (*n* = 28). According to the orthodontic literature with a sample of approximately 26 subjects in each group, the study had a power of at least 80%, to detect a 1.25 standard deviation mean difference between the groups [13].

#### 2.2.1. Damon

Maxillary expansion was obtained with arch wires only, following the Damon treatment philosophy with Damon brackets and Damon Cu-NiTi wide arch wires. All patients finished with .019x.025 TMA or stainless steel upper arch wires. The sample of 23 patients was obtained from a private clinic in Mexico treated between 2015 and 2018.

#### 2.2.2. MARPE

Four self-drilled miniscrews with a length of 7 mm and a diameter of 1.8 mm (ORLUS, Ortholution, Seoul, Korea) were inserted in the palate following local anesthesia. The miniscrews were placed in the center of, and perpendicular to, the 4 mm diameter helical hooks attached to the MARPE appliance. The miniscrews were then connected to the helices using a light-cured resin (Transbond, 3 M Unitek, St Paul, MN, USA) to fix the miniscrews and MARPE appliance together, as well as reduce the potential for irritation to the tongue. The MARPE appliance was activated by a quarter of a turn (0.2 mm) every second day, and expansion was stopped when the palatal cusp of the maxillary first molars came in contact with the buccal cusp tips of the mandibular first molars. The MARPE appliance was then kept for 3 months after active expansion was ceased. Orthodontic treatment with a .022 x.028 inch edgewise straight wire appliances was then commenced. The MARPE sample of 28 patients was treated in Korea for transverse maxillary deficiency at the Department of Orthodontics, Yonsei Dental Hospital, Seoul, Korea, between 2004 and 2010.

#### 2.2.3. PAOO

A full-thickness periosteal flap was reflected, and intentional scoring of both labial and lingual alveolar maxillary cortices was performed. Demineralized freeze-dried bone allograft (DFDBA) or bovine bone xenograft was used to augment the corticotomy sites. The surgical flap was sutured in place, and the patient was seen for orthodontic adjustments every second week after the surgical procedure. The surgical procedure was performed within one week of placement of the orthodontic brackets, and the arch wires were placed and ligated at the time of surgery. PAOO patients were treated with .022 x .028 inch edgewise straight wire appliances until the initial malocclusion was fully resolved. The PAOO adult sample of 28 patients were treated in the private practices of William and Thomas Wilcko (an orthodontist and periodontist, respectively) in Erie, Pennsylvania, USA.

### 2.3. Measurements

Measurements were made on digital STL models utilizing 3Shape Ortho Analyzer 3D scanner software (3Shape, Copenhagen, Denmark) for all 79 subjects in an identical manner. The 3Shape Ortho Analyzer software (3Shape, Copenhagen, Denmark) technique validity has been previously demonstrated [14].

#### 2.3.1. Arch Width

**Figure 1:**
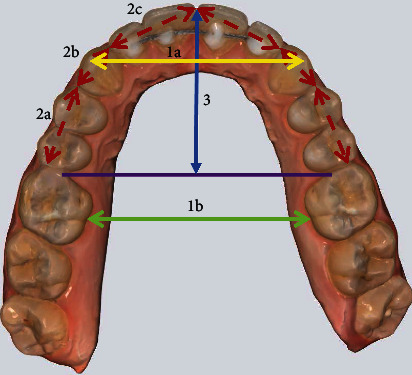
Linear measurements of maxillary dental arch parameters: (1a) inter-canine width from cusp tip to cusp tip; (1b) inter-molar width from mesio-lingual groove at the gingival margin; (2) arch perimeter; and (3) arch depth.

The maxillary transverse arch width was recorded at the level of the canines and first molars. For the inter-canine width, the measurement was made from the cusp tip to cusp tip. For the first molars, the measurement was made from the mesiolingual groove at the gingival margin to the contralateral tooth [15] (Figure 1).

#### 2.3.2. Arch Perimeter

Arch perimeter was measured in three segments per quadrant, starting from the mesial surface of first molars to the mesial surface of first premolars, then from the mesial surface of the first premolars to the mesial surface of the canine, and finally from the mesial surface of the canine to the mesial contact point of the central incisors. The arch perimeter was then calculated by adding the measurements of six segments in each arch [16, 17] (Figure 1).

#### 2.3.3. Arch Depth

Arch depth was determined by measuring a perpendicular line constructed from the mesial contact point of the central incisors to a line connecting the mesial aspect of the first molars. The mesial contact point of the central incisors was determined as the midpoint between the mesial points of the central incisors [16, 17] (Figure 1).

#### 2.3.4. Clinical Crown Height

**Figure 2:**
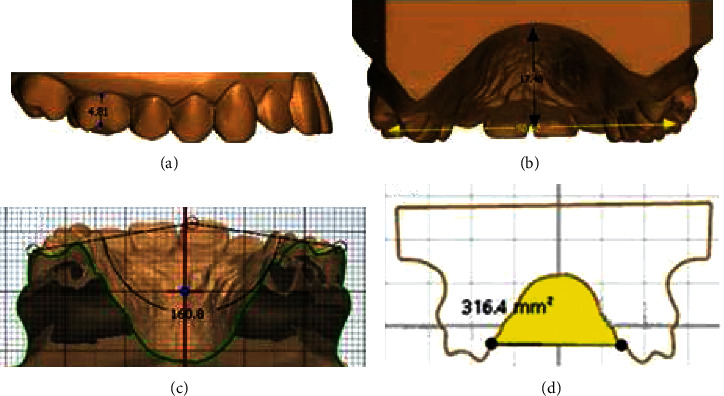
Measurements of maxillary first molar and palate. (a) First molar crown height was determined from the most occlusal point of the buccal groove to the gingival margin below the buccal groove; (b) palatal height was measured at the level of the buccal groove of the first molars with a linear line extended to palate; (c) first molar angulation was determined by measuring the angle of intersection of the lines drawn tangent to the mesio-facial and mesio-palatal cusp tips of the maxillary first molars; and (d) palate vault area measured from the palatal gingival level of the first molars.

The clinical crown height was determined by measuring the distance from the most occlusal point of the buccal groove to the gingival level directly below the buccal groove. This allows for an indirect measure of buccal gingival attachment change from pretreatment to posttreatment [13] (Figure 2(a)).

#### 2.3.5. Palatal Height

The model was cross-sectioned at the plane of the buccal groove of the first molars. A linear line was then dropped to the palatal level, and the height was measured (Figure 2(b)).

#### 2.3.6. Molar Angulations

Molar angulation was determined by measuring the angle of intersection of the lines drawn tangent to the mesio-facial and mesio-palatal cusp tips of the maxillary first molars. Angulation differences between pre- and posttreatment indicate the extent of molar tipping during treatment [18] (Figure 2(c)).

#### 2.3.7. Palatal Vault Area

The palatal vault area was defined as the area superior to the palatal margin of the maxillary first molars [13, 19]. (Figure 2(D)).

### 2.4. Statistical Analysis

To analyze the reliability of the measurements and digital analysis used in this investigation, 10 maxillary dental models were selected randomly and measured twice by a single operator (intraoperator reliability) and then by a second operator (interoperator reliability). The investigator was blinded when performing the measurements. Paired *t*-tests were used to determine intraoperator and interoperator systematic error. The data collected was recorded on a Microsoft Excel Sheet and converted for use with SPSS software (version 20; IBM, Armonk, NY) for data analysis.

A Shapiro-Wilk's test showed that the data was normally distributed; therefore, parametric statistical testing was applied. The mean differences between the pretreatment and posttreatment measurements (increment of change) in each group were evaluated for statistical significance using paired *t*-tests. The mean differences between the three groups were evaluated by analysis of variance (ANOVA) in combination with the Scheffe post hoc test. A *P* value threshold of ≤.05 was accepted as statistically significant.

## 3. Results

The measurement technique used in the study was found to be reliable; repeated measurements on 10 randomly selected study casts demonstrated no significant differences in intra- or interoperator assessments.

At pretreatment, the three groups were heterogeneous for ethnicity, age, and male-female ratios. Mean age of the PAOO (31.7 years) sample was significantly older than the MARPE (20.9 years *P* ≤ .001) sample. Active orthodontic treatment time was significantly shorter for PAOO (8.6 months, *P* ≤ 0.001) than for MARPE and Damon (24.1 and 16.0 months, respectively). There were also more females in the Damon and PAOO groups (64% and 68%, respectively), compared to the MARPE (32%, *P* = 0.19) sample (Table 1).

Heterogeneous (*P* ≤ .05) pretreatment variables were inter-canine width, inter-molar width, and left clinical crown heights. Pretreatment inter-canine width was smaller in PAOO (33.2 mm) compared to Damon (36.1 mm, *P* ≤ .001), and inter-molar width was smaller in PAOO (33.3 mm, *P* ≤ .01) than Damon (36.5 mm) and MARPE (36.4 mm). The left first molar clinical crown height (CCH) for PAOO (4.8 mm) was smaller than MARPE (5.7 mm, *P* ≤ .01) (Table 2). For the three arch parameters that differed significantly at pretreatment, only increments of treatment change were compared among the three study groups. The remaining six arch variables with homogenous pretreatment means were compared at posttreatment in addition to the treatment effect (increments of change) comparisons.

### 3.1. Intergroup Treatment Effects

For the initial variables that were homogenous at pretreatment, posttreatment arch perimeter was significantly greater for MARPE (75.2 mm) at posttreatment than PAOO (72.5 mm, *P* ≤ .05). Arch depth was smaller for MARPE (25.7 mm, *P* ≤ .05) than both Damon (27.1 mm) and PAOO (27.0 mm). Posttreatment right first molar clinical crown height was increased in the MARPE group in comparison to the PAOO group (5.8 vs 5.0 mm, *P* = .01). Palatal vault area was significantly smaller for PAOO (287.6, *P* ≤ .05) than Damon and MARPE (335.5 and 343.1, respectively). There were no significant differences for palatal vault height and molar angulations between the groups (Table 3).

### 3.2. Intergroup Treatment Effects (Increment of Change)

For the initial variables that were heterogeneous at pretreatment, posttreatment inter-canine arch width change was significantly less in Damon (1.4 mm) compared to MARPE (2.3 mm, *P* = .04) and PAOO (3.0 mm, *P* ≤ .001). Inter-molar width increase was larger for MARPE (4.2 mm) than PAOO and Damon (1 and 0.5 mm, respectively, *P* ≤ .001). (Table 4).

### 3.3. Intragroup Treatment Effects

Inter-canine and inter-molar arch widths were significantly increased for all groups posttreatment (*P* ≤ .001). Arch perimeter increased significantly in only the MARPE and PAOO groups (*P* ≤ .001). Clinical crown height for MARPE increased significantly for the right first molar. Palatal vault height for MARPE significantly decreased, and palatal vault area for MARPE increased significantly. PAOO demonstrated a significant increase in first molar angulations (Table 5).

## 4. Discussion

The three groups compared were heterogeneous for ethnicity, age, and male-female ratios as well as total treatment time. The MARPE group was considerably younger, with a mean age of 20.9, which seems to be around the ideal age to attempt MARPE, i.e., after sutural closure, but prior to maturation [20]. Treatment using PAOO was completed within 9 months, which is purported to be due to the Regional Acceleratory Phenomenon (RAP) [8, 10, 21, 22]. Damon showed a swift 16-month treatment time, while patients treated with MARPE completed the treatment in a slower 24-month period. Treatment time using MARPE could have been slower due to the dual phase nature of the treatment, in which the first stage utilized the MARPE appliance itself and the second phase utilized the conventional fixed appliance. The treatment of the MARPE group also took place in an academic setting, unlike the former which were treated privately, thereby perhaps extending the treatment time as well. Therefore, not too much can be read into these differences due to the heterogonous collection of the sample, and the differing protocols by the various practitioners. Three pretreatment dental arch parameters also differed, i.e., left clinical crown height and arch widths at the inter-canine and inter-molar levels. In treatment effect studies, a study design with statistically homogeneous initial means is optimal. Because three arch parameters differed significantly at pretreatment, intergroup posttreatment means were not compared for these three study variables. However, statistical comparisons were made among the three samples for the remaining six maxillary dental arch variables.

Posttreatment, MARPE therapy had a greater impact on arch perimeter than PAOO resulting in 2.7 mm greater arch-perimeter than PAOO-treated patients. However, the posttreatment arch depth was smaller for MARPE (25.7 mm) than both Damon (27.1 mm) and PAOO (27.0 mm). The likely explanation for these two results is that the 4.5 mm average inter-molar width increase with MARPE caused the initial long, narrow arches to expand significantly, thus normalizing the arch form and reducing the arch depth after space closure. In the Damon and PAOO groups, the expansion would seem to be inadequate to cause a significant change in arch form and hence arch depth.

The palatal vault area was significantly smaller at posttreatment for PAOO (287.6) than Damon and MARPE (335.5 and 343.1, respectively). This result is explained by the surgical addition of the bone graft placed palatally in the PAOO group during the procedure, thereby reducing palatal area at the first molar level. Posttreatment right first molar clinical crown height was greater for MARPE than PAOO (5.8 vs 5.0 mm). The MARPE design in this study utilized bands on the upper first molars; therefore, some force was placed on the dentition during the expansion. This force on the upper first molars may have caused some detrimental effects on the periodontium. PAOO, on the other hand, had reduced clinical crown height perhaps due to the alveolar bone graft placed labially resulting in a more robust periodontium.

The increments of change of initially heterogeneous variables showed that the inter-canine expansion obtained was significantly less in the Damon (1.4 mm) treatment system compared to MARPE (2.3 mm) and PAOO (2.8 mm) and also confirms the results of a previous study comparing conventional RME and Damon [23]. Similarly, inter-molar expansion with MARPE (4.3 mm) dramatically exceeded Damon (0.5 mm) and PAOO (1.0 mm), i.e., inter-molar expansion with MARPE was over 8-times greater than Damon and over 4-times greater than PAOO. The inter-molar changes clearly demonstrate the superiority of skeletal expansion caused by sutural separation from the MARPE appliance compared to the limited dental arch wire expansion from both the Damon and PAOO group. Arch wire expansion seems to expand the inter-canine width much more than the inter-molar area, which may result in reduced long-term stability. Surprisingly, the PAOO group showed slightly greater expansion in the canine area compared to the MARPE group.

Intragroup results show that arch depth and left clinical crown height did not significant change within any of the three study groups. All three study groups demonstrated significant inter-molar and inter-canine expansion. In the Damon group, none of the seven remaining arch variables changed significantly during treatment. Within the MARPE group, arch perimeter increased (2.5 mm), palatal height decreased significantly (-0.5 mm), but palatal vault area increased (19.8). The use of miniscrews likely prevented the maxillary first molars from extruding, and the 4.5 mm expansion would explain the increase in palatal vault area in the maxillary first molar region. The arch perimeter also increased within the PAOO (1.2 mm) group, and the 2.5 degree increase in molar angulation would suggest that molar expansion with arch wires resulted in some buccal tipping of the crowns of the first molars. All intragroup statistically significant changes also exceeded clinically significant guidelines except for the inter first molar expansion using the Damon bracket system treatment which was only marginally clinically significant, i.e., 0.5 mm.

### 4.1. Limitations

The retrospective nature of the study and the heterogeneous groups mean that the results of this study should be construed with some caution. The Damon sample was obtained from Mexico, the MARPE sample was from South Korea, and the PAOO sample was obtained from the USA. All patients treated by expansion were followed by straight wire treatment mechanics, with various finishing wires and arch forms that may have impacted some results. Moreover, impressions were taken immediately posttreatment, and gingival inflammation and/or gingiva compression during alginate impression may have affected the STL models. This study was based only on model evaluation, and CBCT was not performed, and thus skeletal changes could not be investigated.

## 5. Conclusions

- All groups showed significant width increase in the canine and molar area
- MARPE obtained significantly greater amount of posterior expansion (4.2 mm) compared to PAOO (1 mm) and Damon (0.5 mm)
- MARPE and PAOO showed significantly greater expansion in the canine area (2.3 mm and 3 mm, respectively) compared to Damon (1.4 mm)
- MARPE was the only group to show a significant increase in palatal vault area

## Figures and Tables

**Table 1:** Demographics representing the three study samples including sample size, mean age (in years), gender (number and percent of sample), and active orthodontic treatment time (in months). Note significant differences (∗*P* ≤ .05) in PAOO age, male and female ratio for MARPE, and active treatment time for PAOO.

Variable	Damon	MARPE	PAOO
Sample size	23	28	28
Mean age (in years)	25.6	20.9	31.7∗
Gender			
Male	8 (35%)	19 (68%)∗	9 (32%)
Female	15 (65%)	9 (32%)∗	19 (68%)
Active Tx (months)	16.0 ± 2.0	24.1 ± 9.3	8.6 ± 3.2∗

**Table 2:** Heterogeneous pretreatment maxillary dental arch variables among the three study samples. Note that the PAOO sample had significantly (∗) smaller (*P* ≤ .006) mean pretreatment dimensions for inter-canine and inter-molar widths as well as left first molar clinical crown height (CCH).

	Initial mean			P sig.		
	Damon	MARPE	PAOO	D-M	D-P	M-P
Inter-canine width	36.1	34.6	33.2	NS	≤.001∗	NS
Inter-molar width	36.5	36.4	33.3	NS	≤.01∗	≤.01∗
Left CCH	5.2	5.7	4.8	NS	NS	≤.01∗

**Table 3:** A comparison among the three study samples of posttreatment variables that were homogeneous at pretreatment.

	Post Tx mean			P sig.		
	Damon	MARPE	PAOO	D-M	D-P	M-P
Arch perimeter	74.5	75.2	72,5	NS	NS	≤.05∗
Arch depth	27.1	25.7	27	≤.05∗	NS	≤.01∗
Right CCH	5.3	5.8	5	NS	NS	≤.01∗
Palatal vault height	18.6	18.4	17.1	NS	NS	NS
Palatal vault area	335.5	343.1	287.6	NS	≤.05∗	≤.01∗
Molar angulation	158	162	160.4	NS	NS	NS

**Table 4:** An intergroup comparison of mean increments of treatment change for pretreatment variables that were heterogeneous.

	Mean change from pre- to posttreatment								
	Damon	MARPE	PAOO	D-M		D-P		M-P	
				Mean dif.	P sig.	Mean dif.	P sig.	Mean dif.	P sig.
Inter-canine width	1.4	2.3	2.8	-0.9	≤0.05	-1.4	≤0.01	-0.5	NS
Inter-molar width	0.5	4.2	1.0	-3.6	≤0.01	-0.5	NS	3.2	≤0.01

**Table 5:** Paired *t*-tests demonstrated pre- to posttreatment intragroup treatment changes.

Variable	Damon group *n* = 23 (8 M; 15F)			MARPE group *n* = 28 (19 M; 9F)			PAOO group *n* = 28 (9 M; 19F)		
	Mean change	SD	*P* signif	Mean change	SD	*P* signif	Mean change	SD	*P* signif
Inter-canine width	1.4	1.66	≤.001∗	2.3	1.21	≤.001∗	3.0	0.76	≤.001∗
Inter-molar width	0.5	1.21	.048∗	4.2	1.87	≤.001∗	1.0	0.72	≤.001∗
Arch perimeter	1.6	4.38	NS	2.5	2.55	≤.001∗	1.2	1.53	≤.001∗
Arch depth	0.4	2.16	NS	-0.1	2.05	NS	0.3	2.25	NS
Right clinical crown height	0.2	0.60	NS	0.3	0.51	.003∗	-0.1	0.90	NS
Left clinical crown height	0.0	0.49	NS	0.2	0.43	NS	-0.1	0.63	NS
Palatal vault height	0.4	1.20	NS	-0.5	1.18	.025∗	-0.2	-0.91	NS
Palatal vault area	12.9	30.6	NS	19.8	35.36	.006∗	-6.8	18.26	NS
Molar angulations	-3.9	9.34	NS	-1.9	8.97	NS	2.5	6.14	.040∗

## Data Availability

The datasets used and/or analyzed during the current study are available from the corresponding author on reasonable request.
